# Implausible, not impossible: delayed supradiaphragmatic thoracic migration of a ventriculoperitoneal shunt in a 17-month-old

**DOI:** 10.1007/s00381-024-06670-8

**Published:** 2024-12-06

**Authors:** Ioan-Alexandru Florian, Paula Topal, Teodora-Larisa Florian, Dragos Font, Ioan-Stefan Florian

**Affiliations:** 1 Clinic of Neurosurgery, Cluj County Emergency Clinical Hospital, 400012 Cluj-Napoca, Romania; 2 Department of Maxillofacial Surgery and Radiology, Faculty of Dental Medicine, Iuliu Hatieganu University of Medicine and Pharmacy, 400006 Cluj-Napoca, Romania; 3 Department of Neurosciences, Iuliu Hatieganu” University of Medicine and Pharmacy, 400012 Cluj-Napoca, Romania; 4 Department of Physiology, Iuliu Hatieganu” University of Medicine and Pharmacy, 400006 Cluj-Napoca, Romania; 5 Faculty of Medicine, “Iuliu Hatieganu” University of Medicine and Pharmacy, 400006 Cluj-Napoca, Romania

**Keywords:** Ventriculoperitoneal shunt (VPS), Migration, Thoracic, Thoracostomy, Hydrothorax, Dyspnea, Supradiaphragmatic

## Abstract

**Background:**

Intrathoracic migration of a ventriculoperitoneal shunt (VPS) is a phenomenally rare complication, with the supradiaphragmatic intercostal variant even more so. Whereas it can prove debilitating or even fatal via massive hydrothorax, the causative mechanism and proper management of this occurrence are undefined.

**Case presentation:**

A 17-month-old girl who had undergone VPS insertion at one month of age was brought to our department for somnolence and dyspnea, which had a sudden onset. Despite a previous thoracostomy provided temporary symptom relief, she had relapsed. Computed tomography (CT) of the chest showed a large loop of the right-sided VPS penetrating into the thorax through the anterior wall, as well as marked right hydrothorax. She was subjected to VPS revision and thoracostomy, with the swift, complete, and lasting remission of her complaints.

**Conclusion:**

It is possible that local reaction coupled with negative inspiratory pressure caused the catheter loop to break into the pleural cavity. Our case demonstrates an exceedingly rare event that has a favorable prognosis if diagnosed and treated quickly and appropriately.

## Introduction

Ventriculoperitoneal shunts (VPS) represent the most widely used procedure for diverting cerebrospinal fluid (CSF) from the cerebral ventricles in case of hydrocephalus [29]. As the name entails, the procedure consists of a catheter that connects the ventricles with the highly absorptive peritoneal cavity. It is indicated for hydrocephalus arising from all manner of pathologies, either congenital or acquired, and has been performed since the turn of the twentieth century [29, 31]. Another widespread procedure for hydrocephalus treatment is the endoscopic third ventriculocisternostomy (ETV), creating a breach within the floor of the third ventricle that allows the passage of CSF directly into the basal cisterns. However, it is not recommended for infants under 3 months of age due to the high failure rate [30].

Congenital hydrocephalus is the excessive and symptomatic buildup of CSF within the ventricles and is present from birth or shortly thereafter [14]. It is more difficult to treat than adult hydrocephalus and is significantly correlated with a higher prevalence of cognitive and developmental deficiencies [26]. Several etiologies may lead to the development of this multifactorial condition, such as primary Sylvian aqueduct stenosis, germinal matrix hemorrhage, intrauterine infections and subsequent gliosis, neural tube defects, exposure to teratogenic substances, nutritional anomalies, X-linked hydrocephalus, to name a few. The proteomic and metabolic profiles of CSF are profoundly altered in congenital hydrocephalus, although our understanding in this field is still in its infancy [23].

Migration of the VPS catheter is a possible complication of this procedure, most commonly as a result of local inflammation, infection, or improper tunneling while inserting the shunt [5]. It can occur either in the internal organs or cavities, most commonly for adults, or externally, an incident more frequently described in infants [3]. Intrathoracic migration of the distal end is, however, extremely rare and the mechanism leading to this circumstance is uncertain [11]. Based on the site of catheter displacement, there are two types of thoracic migration: transdiaphragmatic and supradiaphragmatic [22].

Herein, we present the case of a toddler who developed a thoracic migration of the VPS 16 months after it had been placed. We also provide a summative literature review on this phenomenon, as well as our hypothesis on how this transpired.

## Case Report

This 17-month-old girl, who underwent a VPS placement on the right side when she was 28 days old for congenital hydrocephalus, was transferred to our department for shunt repositioning. The initial surgery went without complications, the tube being inserted under tactile guidance by the experienced senior neurosurgeon (ISF) with a blunt passer. The subcutaneous passage was performed caudo-cranially from the abdominal incision, with two counter-incisions: one above the right clavicle, and the second behind the ear to facilitate the insertion of the valve. Her subsequent development was unremarkable. For 2 weeks prior to her second admission 15 months later, she had been treated in another center for somnolence and difficulty breathing. The initial thoracic CT scan performed there showed a large pleural effusion that caused an almost complete atelectasis of the right lung, for which she underwent a tube thoracostomy. After an initial amelioration of her symptoms, her symptoms relapsed and control CT scan revealed the recurrence of the effusion, with a foreign body present within the cavity, suspected to be the VPS catheter.

**Fig. 1:**
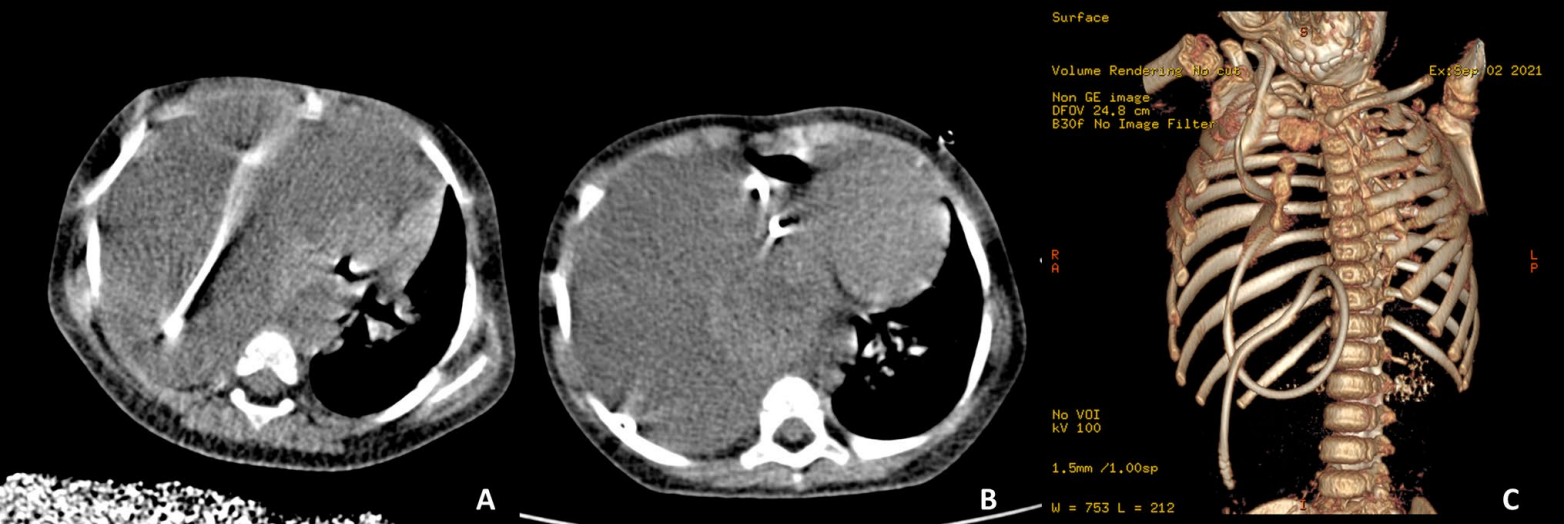
Preoperative chest CT scan demonstrating the massive pleural effusion, with the ventriculoperitoneal catheter visible inside the pleural cavity. **A** Before initial thoracostomy and (**B**) 2 weeks after thoracostomy. **C** 3d reconstruction at 2 weeks after thoracostomy

Upon arriving in our clinic, she was aware, active, slightly somnolent with a pGCS of 15, enlarged cranial perimeter, reactive and symmetrical pupils, present oculocephalic reflex, no visible cranial nerve deficit, present Babinski reflex on both sides, increased muscle tonus, and visible strain while breathing. The cerebral low-radiation dose pediatric CT scan showed a mildly increased ventricular system, with the proper placement of the ventricular end of the shunt within the atrium. However, the thoraco-abdominal CT scan revealed an impressive hydrothorax on the right side with almost complete pulmonary atelectasis, as well as a large hyperintense loop situated in this cavity, which we identified as part of the migrated VPS catheter. The distal end of the shunt was within the abdominal wall, yet completely outside of the peritoneal cavity. 3D reconstruction confirmed the migration of the catheter through the anterior chest wall, through the costal cartilages (Fig. 1). The mother of the patient gave her informed consent on both the revision of the shunt and the publication of this case under anonymity.

She was subjected to shunt repositioning under general anesthesia, with a supraclavicular incision being made on the right side to expose the catheter, while another incision was made on the left paraumbilical area to receive the catheter in the peritoneal cavity. The shunt was isolated from its fibrous sheath and retracted from the thoracic cavity. Via tunneling over the sternum, the two incisions were connected, and the catheter was ultimately placed in the peritoneal cavity on the left side. Simultaneously with this procedure, a right thoracostomy was performed, the fluid being clear white and with no pathological inclusions.

**Fig. 2:**
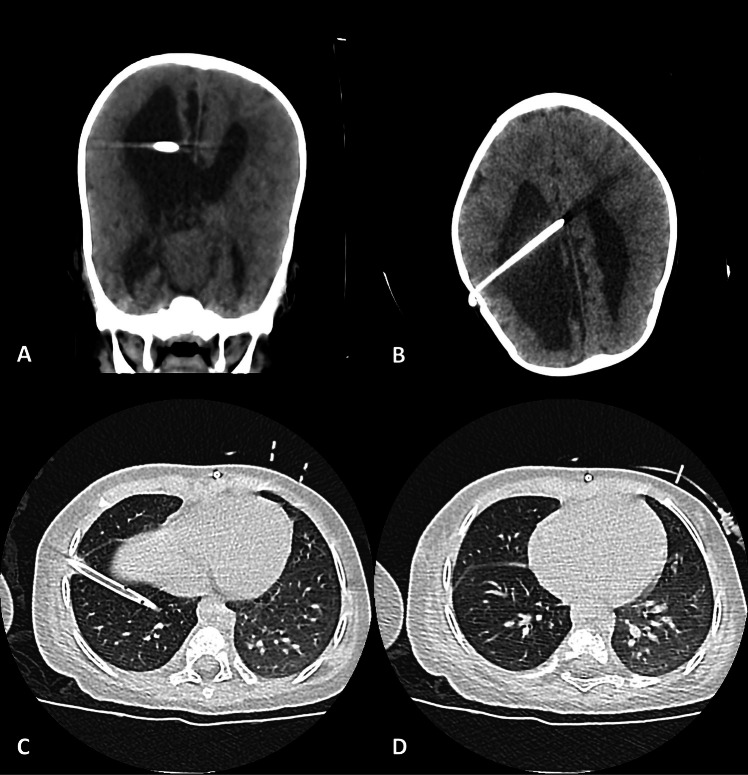
Postoperative CT scan, after the catheter was reinserted over the sternum. **A** Coronal reconstruction and (**B**) axial view of the cranium, demonstrating the placement of the ventricular catheter inside the right ventricle. **C** and **D** Axial chest CT scan showing no remaining pleural effusion, with the thoracostomy drain still in place (visible in C)

Following this intervention, the patient had a complete resolution of her symptoms and respiratory failure. The control CT scan of the head, thorax, and abdomen demonstrated a slight decrease in ventricular diameter, the presence of the thoracostomy drainage in the 7th intercostal space, the lack of fluid within the thoracic cavity and the expansion of the right lung, as well as the proper placement of the distal end at the peritoneal cavity (Fig. 2). The thoracostomy tube remained on water seal and was removed two days after surgery. She was discharged a week later with the recommendation of repeating the CT investigation in case the symptoms recurred. At 3 months follow-up, there were no signs of shunt malfunction or migration, and her neurological status was favorable.

## Discussion and literature review

As was the case for our patient, thoracic migration can lead to somnolence and respiratory failure, yet it can also be asymptomatic in the absence of marked hydrothorax [24]. Aside from dyspnea being the most common presenting symptom, tension hydrothorax, shock symptoms and pleuritic chest pain have also been reported [21]. Differential diagnoses for somnolence and respiratory failure in children with thoracic migration of VPS include valproate toxicity [9, 12, 21], pneumonia [6], or other causes for restrictive lung defects causing dyspnea. According to institutional preference or availability, diagnostic tests for CSF hydrothorax include cell count, biochemical tests, and bacteriological culture from the thoracic fluid, radionuclide shuntogram, beta-2 transferrin, as well as beta-trace protein [9, 12, 21]. However, in our opinion, these should only be considered in case there is no visible migration of the catheter within the thoracic cavity on imaging studies, as to exclude any other probable cause.

Supradiaphragmatic migration of a VPS has been noted to appear most commonly after an erroneous subcutaneous passage while tunneling [22]. Nevertheless, in this setting, the symptoms may arise within days after the intervention and not in a delayed fashion. Blunt subcutaneous passers, as the ones more recently utilized, are much less likely to result in an incorrect tunneling through the intercostal spaces [11]. Yokoya Shigeomi reported a case wherein an incorrect tunneling led to the penetration of the thoracic wall through the intercostal space, arguing that the position of the passer should be confirmed by touch [32]. While this is a reasonable recommendation, especially in adults, in newborns and infants any alteration in the subcutaneous passage may be readily visible. Moreover, the postoperative control thoracic CT can demonstrate an improper subcutaneous trajectory and signs of pneumothorax pertaining to surgical penetration of the pleural cavity [1]. Therefore, the improper placement of the catheter in the initial surgery has been effectively ruled out in our patient. Breaches within the intercostal muscles and pleura can subject the catheter to negative thoracic pressure, ultimately leading to migration [2]. The intercostal space is also more prone to this kind of incident in lean individuals, as is the case for small children or undernourished patients [16]. Such a migration was also reported after VPS placement in a patient who had previously undergone sternotomy [25]. The evidence on hand points to the likelihood that supradiaphragmatic migration can occur under the condition of thinned or weakened intercostal muscles.

However, intrathoracic displacement may also occur through small defects in the diaphragm, a phenomenon known as transdiaphragmatic migration [6, 21]. In this scenario, pleural effusion can ensue even if the catheter is still situated within the peritoneal cavity, particularly if the resorptive capacity of the peritoneum is reduced [9]. This may be accompanied by peritoneal ascites; however, it is not a universal condition [12]. CSF malabsorption is nonetheless a key element for this event, expedited by the reduced surface of the peritoneum in small children. On the other hand, one case of a VPS catheter passing through the liver to finally reach into the lung has also been described [18]. Among the risk factors, a personal history of abdominal infection, previous abdominal surgical interventions, and the development of peritoneal pseudocysts have also been recounted [7, 12]. While it had not been the case for our patient, leakage from the VPS valve may also be a possibility, especially if the valve is not properly secured to the segments of the catheter.

Under general anesthesia, hydrothorax or pleural effusion may lead to an increase in airway pressure coupled with a reduction of dynamic lung compliance and abdominal distension [27]. Pulmonary compliance mirrors the distensibility of the respiratory system, being described as the difference in pressure necessary to expand the lung by a given volume. A safe method of avoiding intraoperative complications related to anesthesia is by closely supervising respiratory system parameters like dynamic lung compliance. It was generally recommended that significant pleural effusions should be drained before the induction of general anesthesia, as these may impede lung expansion, whereas drainage will improve ventilation [8, 15, 20]. However, in the report by Lee et al., general anesthesia could be induced in patients with large pleural effusions before performing thoracostomy and without any deleterious effect on ventilation or cardiovascular functions [8, 15]. However, none of the patients in their report were of pediatric age. Moreover, pulmonary edema arising from post-induction pleural drainage has also been reported [4]. As such, the recommendations and issues concerning the induction of anesthesia in infants and young children with hydrothorax remain elusive.

Regardless of the theories offered, the exact mechanism of catheter thoracic migration is unknown. We hypothesize that, in the case presented, an erosion between the muscle fibers of the intercostal muscles and thin costal cartilages was formed as a reaction to the direct contact with the foreign body represented by the catheter itself. This breach then could have increased in diameter in time as a combined result of compression, local reaction, and muscle contraction while breathing. Once this breach was wide enough, it allowed the slippage of a segment of the catheter, which later formed a loop, slowly and steadily increasing with each respiratory cycle due to inspiratory negative pressure. As the distal end of the shunt was pulled out of the peritoneal cavity and moved further away from the abdomen, the CSF coursed through the tunnel created, yet towards the thoracic cavity, again because of the negative pressure. This could have happened only when the catheter was close enough to the thorax for the alternating intrathoracic pressures to generate the pump effect on the fluid, and sufficiently distant from the abdomen for the CSF not to seep into the peritoneal cavity. However, it cannot be accurately estimated when this phenomenon started occurring in relationship with the onset of the symptoms. Considering that she was symptom-free for 2 weeks following the initial thoracostomy, it is however likely that the phenomenon began in earnest within a month prior to that intervention. Adding to the fact that she did not present any respiratory symptoms until then and that the expansion of the lungs was normal after VPS revision, it can be realistically excluded that the migration had an iatrogenic cause.

Depending on the experience of the treating surgeon, treatment for this complication consists of a VPS repositioning, catheter externalization and ulterior reinternalization [12], switching to an alternative CSF diverting procedure (such as a ventriculoatrial shunt) [9, 10, 19], or the complete removal of the shunt if there is evidence of hydrocephalus resolution [1]. Thoracocentesis or thoracostomy are also recommended, as they result in swift amelioration of the complaints and dyspnea, while the former also provides evidence for the source of hydrothorax [13, 17, 21]. For older patients, especially those with recurrent pleural effusion, positive pressure ventilation through nasal continuous positive airway pressure (nCPAP) may prove a valuable therapeutic tool [28]. We argue that for our patient, since she suffered from congenital hydrocephalus, which implies a lifelong dependency on the shunt, plus the fact that there were no signs of peritoneal malabsorption or bacteriological contamination, our decision to reinsert the distal end into the opposite side of the abdomen was the most suitable.

Highlights of this case report include the young age of the patient, the extremely rare and perplexing complication she sustained, and the timeframe between the surgical placement of the VPS and the occurrence of the supradiaphragmatic migration. Also of note are her fast recovery and complete amelioration of symptoms.

## Conclusions

This case presentation demonstrated a very rare and potentially deadly complication of VPS with an as of yet undetermined causative mechanism. It could prove particularly more dire in infants and toddlers since they possess a smaller thoracic cavity. Respiratory failure in young patients with VPS should exclude the thoracic migration of the catheter, since catheter revision or repositioning is mandatory for the proper management in this scenario.

## Data Availability

No datasets were generated or analysed during the current study.
